# Comparative structural characterization and protective effects against myocardial ischemia/reperfusion injury of polysaccharides from two major Chinese yam cultivars (*Dioscorea opposita* Thunb)

**DOI:** 10.3389/fnut.2026.1864107

**Published:** 2026-07-08

**Authors:** Guojie Xie, Longqin Li, Xueqian Hu, Ang Ma, Yi Ying, Shujie Zhang, Hai-Ning Lyu, Hui Zhao, Xiaoyu Hu, Zipeng Gong, Jigang Wang, Dandan Liu

**Affiliations:** 1 Guizhou Provincial Key Laboratory of Pathogenesis and Drug Research on Common Chronic Diseases, School of Basic Medical Sciences and School of Pharmaceutical Sciences, Guizhou Medical University, Guiyang, China; 2 Department of Oncology, Ningbo Municipal Hospital of Traditional Chinese Medicine Affiliated to Zhejiang Chinese Medical University, Ningbo, China; 3 State Key Laboratory for Quality Assurance and Sustainable Use of Dao-di Herbs, Artemisinin Research Center, Institute of Chinese Materia Medica, China Academy of Chinese Medical Sciences, Beijing, China; 4 Hebei Institute of Industrial Technology for Chinese Medicinal Materials, Anguo, China; 5 School of Traditional Chinese Medicine, Southern Medical University, Guangzhou, China

**Keywords:** Chinese yam, cultivar comparison, functional ingredient, myocardial ischemia/reperfusion injury, polysaccharides, structural characterization

## Abstract

**Background:**

Chinese yam (*Dioscorea opposita* Thunb.) is a widely consumed edible and medicinal crop. Its polysaccharides are promising food-derived bioactive ingredients. However, cultivar-dependent consistency remains insufficiently defined. This issue is important for functional ingredient standardization. In this study, crude polysaccharide fractions isolated from two representative cultivars, Xiao-Bai-Zui and Tie-Gun, were designated CYP-XBZ and CYP-TG and comparatively evaluated.

**Methods:**

The molecular weight distribution, monosaccharide composition, UV–visible spectra, and FT–IR characteristics of the two polysaccharide fractions were compared. Their protective effects were evaluated in H9c2 cells subjected to oxygen–glucose deprivation/reoxygenation (OGD/R) and in a rat model of myocardial ischemia/reperfusion injury (MI/RI). Furthermore, transcriptomics, proteomics, and Western blotting were integrated to investigate the underlying mechanisms.

**Results:**

CYP-XBZ and CYP-TG exhibited broadly similar molecular weight distribution patterns, glucan-dominant monosaccharide compositions, and characteristic polysaccharide spectral features. However, minor differences in molecular heterogeneity and monosaccharide proportions were observed. In OGD/R-treated H9c2 cells, both fractions significantly improved cell viability and reduced CK-MB, cTnT, and 8-OHdG levels. Multi-omics analyses revealed that the shared protective effects were mainly associated with stress and inflammation-related pathways, particularly the MyD88-associated MAPK and NF-κB signaling pathways. These findings were further confirmed by Western blotting. In the rat model of MI/RI, oral administration of both polysaccharide fractions reduced infarct size and alleviated histopathological injury. They also decreased serum biomarkers of myocardial injury and oxidative stress.

**Conclusion:**

Polysaccharides from the two major Chinese yam cultivars showed highly comparable structural features, cardio protective effects, and regulatory mechanisms. These findings support the functional consistency of these cultivars as sources of bioactive polysaccharides and highlight their potential as food-derived functional ingredients for cardiovascular and nutrition-related health.

## 1 Introduction

Yams (*Dioscorea* spp.) are important edible tuber crops that contribute substantially to dietary energy supply and food diversity in many regions of the world (1). Beyond their nutritional role, yams contain a variety of bioactive constituents, including storage proteins, steroidal saponins, phenolic compounds, and non-starch polysaccharides, which have attracted increasing attention for their potential use in functional foods, nutraceuticals, and health-oriented ingredients relevant to metabolic and cardiovascular health (2–4). Among these components, polysaccharides are of particular interest because of their relatively high abundance, structural diversity, and broad bioactivities (5–7).

Chinese yam (*Dioscorea opposita* Thunb.) is a representative edible and medicinal yam species widely consumed in China and other parts of East Asia (2, 8). As a traditional food–medicine homologous resource, it has long been incorporated into daily diets and health-promoting preparations (9, 10). Previous studies have shown that Chinese yam polysaccharides possess antioxidant, immunomodulatory, hypoglycemic, hypolipidemic, and anti-inflammatory activities, supporting their promise as food-derived bioactive ingredients (11–14). However, for the development of such ingredients, biological activity alone is not sufficient; cultivar-related consistency in composition, structure, and function is also essential for reproducible application.

An important issue concerns cultivar-related consistency. For food-derived bioactive ingredients, variation in genotype may influence chemical composition, structural features, and biological performance, which in turn affects the stability, reproducibility, and development potential of functional ingredients (15, 16). In Chinese yam, Tie-Gun is a well-known representative cultivar and has been more extensively studied, whereas Xiao-Bai-Zui is also widely consumed but has received much less comparative and mechanistic attention (17). Whether polysaccharides from these two major edible cultivars exhibit substantial differences in structure and cardio protective activity remains unclear. Clarifying this issue is important not only for understanding the stability of bioactive traits across cultivars but also for supporting the development of Chinese yam as a reliable source of food-derived functional polysaccharides.

In recent years, research on plant polysaccharides has increasingly shifted from simple activity screening toward integrated evaluation of structural features, biological functions, and application potential (18–21). Within this context, cardiovascular health has emerged as an important area for the development of food-derived functional ingredients (22–24). Myocardial ischemia/reperfusion injury (MI/RI), a major pathological event associated with acute myocardial infarction and reperfusion therapy, is characterized by oxidative stress, inflammatory activation, and stress-responsive signaling cascades (25, 26). Among the pathways involved, MAPK and NF-κB signaling are known to play central roles in regulating cellular injury responses and inflammatory processes during myocardial damage (27–30). Accumulating evidence indicates that polysaccharides from edible plants can attenuate ischemia/reperfusion-related injury by modulating oxidative stress and inflammatory signaling (31, 32). Nevertheless, the cardio protective potential of Chinese yam polysaccharides has not been systematically investigated using an integrated *in vitro*/*in vivo* and multi-omics framework.

We hypothesized that the polysaccharides from Xiao-Bai-Zui and Tie-Gun Chinese yams would exhibit largely comparable structural characteristics and cardioprotective activities, with only subtle differences that do not substantially compromise their overall functional conservation. Therefore, in the present study, polysaccharides isolated from Xiao-Bai-Zui and Tie-Gun Chinese yams were comparatively characterized using molecular weight analysis, monosaccharide profiling, and spectroscopic methods. Their protective effects were then evaluated in an oxygen–glucose deprivation/reoxygenation (OGD/R)-induced H9c2 cell injury model and further validated in a rat myocardial ischemia/reperfusion injury (MI/RI) model. To elucidate the underlying mechanisms, transcriptomics, proteomics, and Western blotting validation were integrated to identify shared signaling responses associated with the two polysaccharide fractions. This study aimed to determine whether polysaccharides from two major Chinese yam cultivars exhibit comparable structural and cardio protective features and to provide experimental support for their use as food-derived functional ingredients with potential cardiovascular and nutrition-related health benefits (33).

## 2 Materials and methods

### 2.1 Materials and reagents

Fresh tubers of Tie-Gun yam (*Dioscorea opposita* Thunb, Tie-Gun cultivar) were collected from Wen County, Henan Province, China, while Xiao-Bai-Zui yam (*D. opposita* Thunb, Xiao-Bai-Zui cultivar) was obtained from Anguo County, Hebei Province, China. Both cultivars were harvested during the normal commercial harvest season in autumn 2024. Cultivar identity was confirmed according to local cultivar designation and characteristic tuber morphology. Representative samples were deposited in our laboratory for reference under voucher numbers DO-TG-2024 (Tie-Gun yam) and DO-XBZ-2024 (Xiao-Bai-Zui yam). The tubers were washed, sliced, dried at 50 °C, and pulverized into fine powder before extraction. Monosaccharide reference standards, including arabinose, galactose, glucose, mannose, fructose, rhamnose, ribose, and xylose, were purchased from Shanghai Yuanye Biotechnology Co., Ltd. (Shanghai, China). Unless otherwise specified, all analytical-grade reagents were obtained from Sigma-Aldrich (St. Louis, MO, United States). Chromatographic-grade acetonitrile and methanol were purchased from Thermo Fisher Scientific (Waltham, MA, USA). Ultrapure water was used throughout the study.

### 2.2 Preparation of Chinese yam polysaccharides

Crude polysaccharide fractions from Xiao-Bai-Zui yam and Tie-Gun yam were prepared using the same extraction procedure to ensure comparability and were designated as CYP-XBZ and CYP-TG, respectively. Briefly, yam powder (30 g) was refluxed with 70% ethanol to remove lipophilic constituents and low-molecular-weight compounds. After filtration, the residues were extracted twice with distilled water at a solid-to-liquid ratio of 1:10 (w/v) under reflux for 2 h each. The combined aqueous extracts were concentrated under reduced pressure and precipitated with ethanol to a final concentration of 70% (v/v), followed by incubation at 4 °C overnight. The resulting precipitates were collected by centrifugation at 4000 × g for 10 min, washed with absolute ethanol, and freeze-dried to obtain crude polysaccharide fractions. No further dialysis purification was performed. All physicochemical characterization and biological experiments were performed using crude polysaccharide fractions obtained from a single preparation batch for each cultivar to ensure experimental consistency.

### 2.3 Molecular weight analysis

The molecular weight distributions of CYP-XBZ and CYP-TG were determined by gel permeation chromatography. Each sample (5 mg) was dissolved in 1 mL of distilled water, allowed to stand for 1 h to ensure complete dissolution, and filtered through a 0.22 μm membrane before analysis. Chromatographic separation was performed on a Waters e2696 HPLC system equipped with a refractive index detector and a PL aquagel-OH MIXED-M column (7.5 × 300 mm), maintained at 40 °C. Distilled water was used as the mobile phase at a flow rate of 1.0 mL/min. Linear polyethylene glycol standards were used to construct the calibration curve, and the apparent molecular weights were estimated according to the retention times of the standards.

### 2.4 Monosaccharide composition analysis

Monosaccharide composition was analyzed after acid hydrolysis. Briefly, samples were hydrolyzed with 2 M trifluoroacetic acid at 121 °C for 2 h. After hydrolysis, excess acid was removed under a stream of nitrogen, and the residues were repeatedly washed with methanol before reconstitution in ultrapure water. Monosaccharides were separated using a Thermo ICS 5000 + ion chromatography system equipped with a pulsed amperometric detector and a Dionex CarboPac PA20 column (150 × 3.0 mm, 10 μm; Thermo Fisher Scientific, United States). Individual monosaccharides were identified and quantified by comparison with authentic standards. The monosaccharide composition was expressed as mass percentage of the total identified monosaccharides.

### 2.5 UV–visible and FT–IR spectral analysis

UV spectra of CYP-XBZ and CYP-TG were recorded using a UV–visible spectrophotometer over the range of 190–1,100 nm to evaluate the presence of protein and nucleic acid impurities. Fourier transform infrared spectra were recorded using a Nicolet iS5 FT–IR spectrometer. Dried samples were mixed with spectroscopic-grade KBr, pressed into pellets, and scanned over the range of 4,000–400 cm^−1^ at a resolution of 4 cm^−1^.

### 2.6 Cell culture and OGD/R treatment

H9c2 rat cardiomyoblast cells were cultured in Dulbecco’s modified Eagle’s medium (DMEM; MeilunBio, Dalian, China) supplemented with 10% fetal bovine serum (FBS; MeilunBio) and 1% penicillin–streptomycin. Cells were maintained at 37 °C in a humidified incubator containing 5% CO₂. Cells between passages 5 and 15 were used for all experiments.

When cell confluence reached approximately 70%–80%, cells were treated with CYP-XBZ or CYP-TG at final concentrations of 25, 50, or 100 μg/mL for 24 h. These concentrations were selected based on preliminary cytotoxicity testing, which showed no obvious reduction in basal cell viability at concentrations up to 1,000 μg/mL, and on commonly used *in vitro* concentration ranges for food-derived polysaccharides. Oxygen–glucose deprivation was induced by replacing the culture medium with glucose-free DMEM and transferring the cells to an anaerobic chamber containing 95% N₂ and 5% CO₂ for 2 h. Reoxygenation was initiated by restoring normoxic conditions and culturing the cells in complete DMEM for 24 h. The 2 h OGD/24 h reoxygenation protocol was selected because it produced stable and reproducible injury in preliminary optimization while preserving sufficient cell viability for intervention assessment. A control group was cultured in normal DMEM under normoxic conditions without OGD/R treatment.

### 2.7 Cell viability assay

H9c2 cells were seeded into 96-well plates at 6,000 cells per well and allowed to adhere for 24 h. After treatment, cell viability was assessed using a Cell Counting Kit-8 (CCK-8; MeilunBio) according to the manufacturer’s instructions. Briefly, 10 μL of CCK-8 solution was added to each well and incubated at 37 °C for 1 h. Absorbance was measured at 450 nm using a microplate reader (BioTek Instruments, Winooski, VT, United States). Cell viability was expressed relative to that of the control group.

### 2.8 Animals and experimental design

Male Sprague–Dawley (SD) rats (8 weeks old, 200–220 g) were obtained from an accredited laboratory animal center and housed under standard conditions (22 ± 2 °C, 12 h light/dark cycle, and 50%–60% relative humidity) with free access to standard chow and water. All animal procedures were approved by the Animal Experimental Ethics Review Committee of the Institute of Basic Research in Chinese Medicine, China Academy of Chinese Medical Sciences (IBTCMCACMS21-2503-10), and were conducted in accordance with the National Institutes of Health Guide for the Care and Use of Laboratory Animals. Rats were purchased from Si Bei Fu (Beijing) Laboratory Animal Technology Co., Ltd. (SCXK (Beijing) 2024–0001).

After 1 week of acclimatization, the rats were randomly assigned to four groups, with 15 rats in each group: sham, model, CYP-XBZ, and CYP-TG groups. Rats in the sham and model groups received an equal volume of physiological saline. CYP-XBZ and CYP-TG were dissolved in physiological saline and administered by oral gavage at 100 mg/kg once daily for 7 consecutive days before surgery. The animal dose was selected based on pilot experiments and a commonly used dose range for food-derived polysaccharides in rodent studies (50–200 mg/kg). Rats in the model group and the two yam polysaccharide-treated groups underwent ligation of the left anterior descending (LAD) coronary artery for 30 min followed by reperfusion, and rats in the sham-operated group underwent the same surgical procedure without ligation. An additional dose of CYP-XBZ or CYP-TG was administered before reperfusion. Blood and tissue samples were collected 24 h after reperfusion. Animals used for tissue collection were deeply anesthetized with pentobarbital sodium (50 mg/kg, intraperitoneally) before sample collection. No animals were excluded from the final analysis. The final sample sizes were *n* = 6 per group for infarction volume and serum biomarker analyses and *n* = 3 per group for histological and Western blotting analyses. At the end of the experiment, all remaining animals were euthanized by carbon dioxide inhalation using a gradual-fill method, with CO₂ introduced at a displacement rate of approximately 30% of the chamber volume per minute. Death was confirmed by cessation of heartbeat and respiration. Some tissues were preserved as specimens, while the carcasses were temporarily stored in a freezer at the experimental center and later transferred to a professional disposal company for unified treatment. All efforts were made to minimize animal suffering and reduce the number of animals used.

### 2.9 Rat MI/RI model

MI/RI was established by transient ligation of the LAD coronary artery with minor modifications to previously described procedures (34, 35). Briefly, rats were anesthetized with pentobarbital sodium (50 mg/kg, intraperitoneally), followed by endotracheal intubation and mechanical ventilation using a small-animal ventilator. A left thoracotomy was performed through the fourth intercostal space to expose the heart. The left anterior descending coronary artery was ligated approximately 2–3 mm from its origin using a 6–0 polypropylene suture. Successful ischemia was confirmed by blanching of the anterior ventricular wall and ST-segment elevation on electrocardiography. After 30 min of ischemia, the ligature was released to allow reperfusion. Sham-operated rats underwent the same surgical procedure without coronary ligation. Blood and heart tissue samples were collected 24 h after reperfusion for subsequent analyses.

### 2.10 Determination of cardiac injury and oxidative stress markers

Creatine kinase-MB (CK-MB), cardiac troponin T (cTnT), and 8-hydroxy-2′-deoxyguanosine (8-OHdG) were measured using commercial assay kits (CK-MB(MM-0625R1), CTnT(MM-0795R1), 8-ohdg (MM-0224R1) respectively Jiangsu Meimian Industrial Co., Ltd) according to the manufacturers’ instructions. In the cell experiments, culture supernatants were used for CK-MB and cTnT measurements, whereas cell lysates were used for 8-OHdG analysis. Total protein concentrations were determined using a bicinchoninic acid assay (BCA) kit (Jiangsu Cowin Biotech Co., Ltd) for normalization where applicable.

### 2.11 Electrocardiographic (ECG) monitoring

Lead II electrocardiograms were continuously recorded during surgery using a biological signal acquisition system. After anesthesia and endotracheal intubation, needle electrodes were inserted subcutaneously into the limbs to ensure stable signal acquisition. A 10 min baseline ECG trace was recorded before ischemia induction.

### 2.12 TTC staining

After 24 h of reperfusion, rats were euthanized and the hearts were rapidly excised and rinsed with ice-cold saline to remove residual blood. The hearts were pre-frozen at −80 °C for 10 min and transversely sliced into six sections of 1–2 mm thickness. The sections were incubated in 2% 2,3,5-triphenyltetrazolium chloride (TTC) solution at 37 °C for 15 min in the dark and then fixed in 4% paraformaldehyde. Viable myocardium was stained brick red, whereas infarcted tissue remained pale white. Images were analyzed using ImageJ software by investigators blinded to group allocation. Infarct size was expressed as the percentage of infarcted area relative to the total ventricular area.

### 2.13 Hematoxylin and eosin (H&E) staining

Rat hearts were fixed in 4% paraformaldehyde, dehydrated through graded ethanol series, embedded in paraffin, and sectioned at 5 μm thickness. The sections were stained with H&E to assess myocardial morphology. Histopathological changes were observed under a light microscope. Myocardial injury was evaluated using a semi quantitative scoring system ranging from 0 to 3, where 0 indicated no detectable damage and 3 indicated severe structural disruption. Histopathological scoring was performed by investigators blinded to group allocation.

### 2.14 Masson’s trichrome staining

Rat’s myocardial sections were stained with Masson’s trichrome to evaluate collagen deposition and fibrosis. After deparaffinization and rehydration, the sections were treated with Bouin’s solution at 56 °C for 1 h, stained with Weigert’s iron hematoxylin, and counterstained with acid fuchsin and aniline blue. Collagen fibers were stained blue and viable myocardium was stained red. Images were captured under a light microscope, and the collagen volume fraction was quantified using ImageJ software as the ratio of collagen-stained area to total myocardial area, and quantification was performed by investigators blinded to group allocation.

### 2.15 Transcriptomic analysis

Total RNA was extracted from cells in the model, CYP-XBZ, and CYP-TG groups and subjected to RNA sequencing on an Illumina platform. Differentially expressed genes (DEGs) were identified for the pairwise comparisons CYP-XBZ versus model and CYP-TG versus model using the criteria |log₂ fold change| > 1 and adjusted *p* < 0.05. This threshold was selected to identify genes with both statistically significant and biologically meaningful transcriptional changes, considering the relatively broad dynamic range of RNA-seq data. Independent cell samples were used as biological replicates for transcriptomic analysis. Volcano plots were generated to visualize the distribution of DEGs. Functional enrichment analysis was performed using the Kyoto Encyclopedia of Genes and Genomes (KEGG) database. Gene set enrichment analysis (GSEA) was used to evaluate coordinated pathway-level changes. Shared DEGs were identified by Venn analysis and subsequently subjected to KEGG enrichment analysis.

### 2.16 Proteomic analysis

Proteomic profiling was performed using a Thermo Scientific Orbitrap Fusion Lumos mass spectrometer. Protein identification and quantification were carried out using Proteome Discoverer version 2.4 against the appropriate UniProt database. Differentially expressed proteins (DEPs) were screened using the criteria |log₂ fold change| > 0.26 and adjusted *p* < 0.05 for the comparisons model versus control, CYP-XBZ versus model, and CYP-TG versus model. A lower fold-change cutoff was applied for proteomic screening because mass spectrometry-based protein quantification generally has a narrower dynamic range and lower detection sensitivity than transcriptomic analysis; therefore, statistical significance was combined with a moderate fold-change threshold to retain biologically relevant protein-level changes. Independent cell protein samples were used as biological replicates for proteomic analysis. Volcano plots were used to visualize the distribution of DEPs. KEGG pathway enrichment analysis was performed to evaluate functional implications. Shared DEPs were identified to generate an intersection protein set for further enrichment analysis. Protein–protein interaction (PPI) networks were constructed using publicly available interaction databases, with node size and color intensity representing degree centrality.

### 2.17 Western blotting analysis

Total protein was extracted using RIPA lysis buffer (Applygen, C1053) supplemented with protease and phosphatase inhibitors. Protein concentrations were determined using a BCA kit. Equal amounts of protein were separated by SDS-PAGE and transferred onto PVDF membranes. After blocking, the membranes were incubated overnight at 4 °C with primary antibodies against p-NF-κB P65 (Ser536), NF-κB P65, p-P38 MAPK (Thr180/Tyr182), P38 MAPK, CEBPB, and MyD88 at a dilution of 1:1000. After washing with TBST, the membranes were incubated with horseradish peroxidase-conjugated secondary antibodies. Protein bands were visualized using an enhanced chemiluminescence kit (MeilunBio, MA0186), imaged with a Syngene GeneGnome XRQ system, and quantified using GeneTools software. Western blotting experiments were performed using independent biological samples, and representative blots and quantitative analyses were derived from the same set of biological replicates.

### 2.18 Statistical analysis

All experiments were independently repeated at least three times. For cell-based assays, biological replicates represented independent cell culture experiments or independently treated wells, as appropriate. For animal experiments, each animal was considered one biological replicate. For transcriptomic, proteomic, and Western blotting analyses, biological replicates represented independently collected cell or tissue samples. Data are presented as mean ± SEM. Statistical analyses were performed using SPSS version 27.0 (IBM Corp., Armonk, NY, United States). Differences among multiple groups were assessed by one-way analysis of variance followed by Tukey’s *post hoc* test. A value of *p* < 0.05 was considered statistically significant. Exact *p* values were provided where feasible in the source data or text; otherwise, statistical significance is indicated using the symbols defined in the corresponding figure legends. Graphs were generated using GraphPad Prism 9.0 and OriginPro 2023. Transcriptomic and proteomic visualizations were generated using established bioinformatics platforms, including Hiplot and ImageGP.

## 3 Results

### 3.1 Preparation of CYP-XBZ and CYP-TG

**Figure 1:**
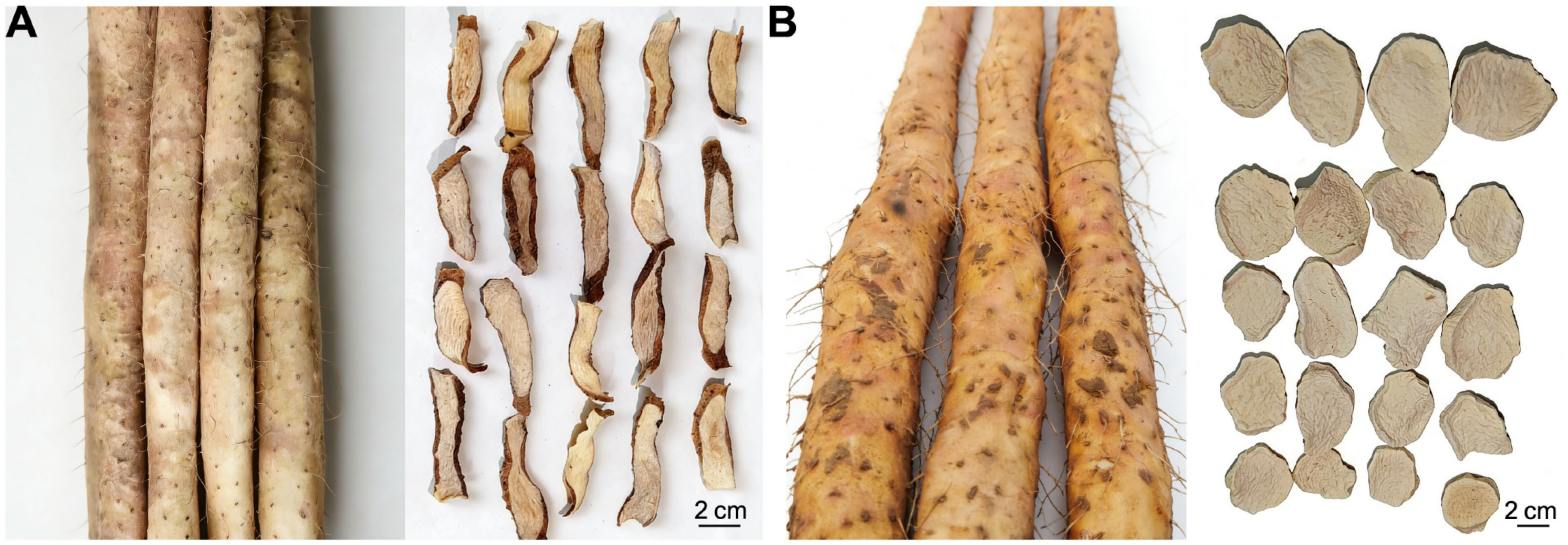
Morphological characteristics of two widely consumed edible cultivars of Chinese yam (*D. opposita* Thunb.). Representative photographs of whole tubers and transverse sections of Xiao-Bai-Zui **(A)** and Tie-Gun **(B)**.

The extraction of plant polysaccharides is influenced by processing parameters such as temperature, extraction time, and solvent system. These factors can affect both yield and structural integrity. Previous studies have shown that optimization of aqueous extraction conditions is necessary to ensure reproducibility and to preserve biological activity. In this study, an identical hot-water extraction protocol was applied to Tie-Gun yam and Xiao-Bai-Zui yam to allow direct comparison. This approach was selected because it is simple, scalable, and suitable for maintaining native structural features. Based on a modified procedure from previous reports, crude polysaccharides were extracted under optimized aqueous conditions. The extracts were then concentrated and precipitated to obtain the final fractions. The extraction yield of CYP-XBZ was 7.5%, while that of CYP-TG was 7.4%. The nearly identical yields indicate comparable extraction efficiency under the same experimental conditions. Representative photographs of the whole tubers and transverse sections of the two cultivars are shown in Figures 1A,B.

### 3.2 Chemical characterization of CYP-XBZ and CYP-TG

**Figure 2:**
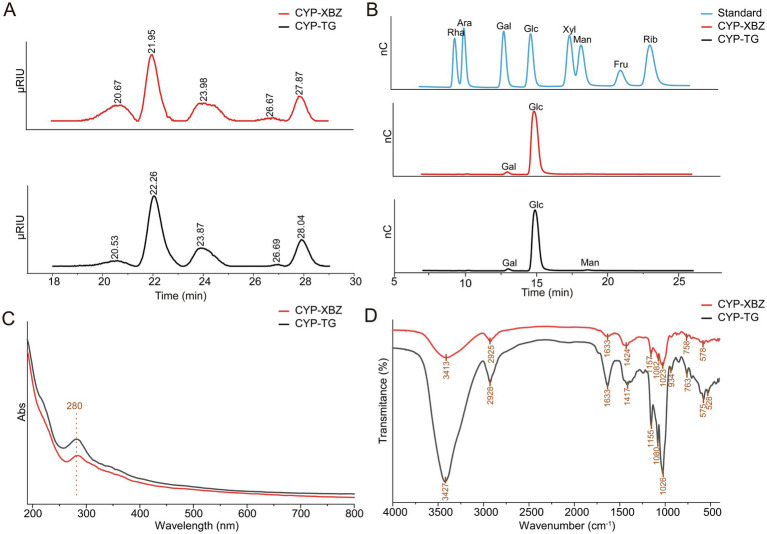
Chemical characterization of CYP-XBZ and CYP-TG. **(A)** GPC profiles. **(B)** Monosaccharide composition. **(C)** UV–visible spectra. **(D)** FT–IR spectra.

To characterize the physicochemical properties of the two polysaccharide fractions, molecular weight distribution, monosaccharide composition, and spectroscopic features were analyzed (Table 1, Figure 2).

**Table 1:** Molecular weight distribution of CYP-XBZ and CYP-TG.

CYPs	Retention times (min)	Number average molecular weight (Da)	Weight average molecular weight (Da)
CYP-XBZ	20.67	3.71 × 10^5^	3.91 × 10^5^
	21.95	1.72 × 10^5^	1.75 × 10^5^
	23.98	5.59 × 10^4^	5.83 × 10^4^
	26.67	1.23 × 10^4^	1.27 × 10^4^
	27.87	4.98 × 10^3^	5.12 × 10^3^
CYP-TG	20.53	4.06 × 10^5^	4.32 × 10^5^
	22.26	1.67 × 10^5^	1.70 × 10^5^
	23.87	6.13 × 10^4^	6.33 × 10^4^
	26.69	1.07 × 10^4^	1.08 × 10^4^
	28.04	4.84 × 10^3^	5.03 × 10^3^

#### 3.2.1 Molecular weight distribution

Molecular weight distribution is an important structural parameter that influences physicochemical behavior and bioactivity. As shown in Figure 2A and Table 1, both CYP-XBZ and CYP-TG displayed multiple elution peaks in GPC chromatograms. This pattern indicates heterogeneous polysaccharide populations rather than a single homogeneous species. CYP-XBZ exhibited five distinguishable fractions with Mw values of approximately 3.91 × 10^5^, 1.75 × 10^5^, 5.83 × 10^4^, 1.27 × 10^4^, and 5.12 × 10^3^ Da. CYP-TG also showed five fractions, with Mw values of 4.32 × 10^5^, 1.70 × 10^5^, 6.33 × 10^4^, 1.08 × 10^4^, and 5.03 × 10^3^ Da. The overall molecular weight ranges were similar in both samples, spanning approximately 5 × 10^3^ to more than 4 × 10^5^ Da. Slight differences were observed in individual fractions. The highest molecular weight component of CYP-TG was marginally higher than that of CYP-XBZ. In contrast, CYP-XBZ showed a slightly greater Mw in the mid–low molecular weight region. Minor shifts in retention times were also detected (e.g., 21.95 vs. 22.26 min; 27.87 vs. 28.04 min). These shifts suggest small differences in hydrodynamic volume and molecular heterogeneity. Overall, the molecular profiles of the two cultivars were comparable, with modest variation in distribution patterns.

#### 3.2.2 Monosaccharide composition

Monosaccharide analysis showed that both CYP-XBZ and CYP-TG are neutral heteropolysaccharides primarily composed of glucose (Table 2, Figure 2B). Glucose accounted for 97.4% of total monosaccharides in CYP-XBZ and 96.8% in CYP-TG. Galactose was detected at low levels (2.6 and 2.2%, respectively). Mannose was present at 1.0% only in CYP-TG. No uronic acids were detected under the analytical conditions used. The chromatographic profiles of the two samples were nearly overlapping, and the qualitative monosaccharide species were identical. Differences were limited to small quantitative variations in relative proportions. These data indicate that the primary carbohydrate backbone composition is largely conserved between the two cultivars.

**Table 2:** Monosaccharide composition of CYP-XBZ and CYP-TG (MR).

Monosaccharides	CYP-XBZ	CYP-TG
Gal (%)	2.6	2.2
Glc (%)	97.4	96.8
Man (%)	0	1.0

#### 3.2.3 UV–visible spectral analysis

The UV–visible spectra of CYP-XBZ and CYP-TG are shown in Figure 2C. Both samples exhibited strong absorption in the low-wavelength region around 200 nm, which is typical for polysaccharides. No absorption peak was observed at 260 nm, indicating the absence of nucleic acid contamination. A weak signal near 280 nm was detected in both samples, suggesting trace protein residues. The spectral profiles were nearly identical, indicating similar purity and effective removal of nucleic acids and most protein components.

#### 3.2.4 FT–IR spectral characteristics

FT–IR spectra of CYP-XBZ and CYP-TG are presented in Figure 2D. Both samples showed typical polysaccharide absorption bands. A broad peak around 3,400 cm^−1^ corresponds to O–H stretching vibrations, reflecting abundant hydroxyl groups and hydrogen bonding. Weak bands near 2,920 cm^−1^ are attributed to C–H stretching vibrations. Signals in the 1,600–1,650 cm^−1^ region may arise from bound water bending vibrations or minor protein residues, consistent with UV observations. Strong absorption bands between 1,000 and 1,150 cm^−1^ correspond to C–O–C and C–O stretching vibrations, indicating glycosidic linkages and pyranose structures. The FT-IR spectra of both fractions were highly similar in peak position and pattern, demonstrating comparable core structural features.

### 3.3 Protective effects of CYP-XBZ and CYP-TG against OGD/R-induced injury in H9c2 cells

**Figure 3:**
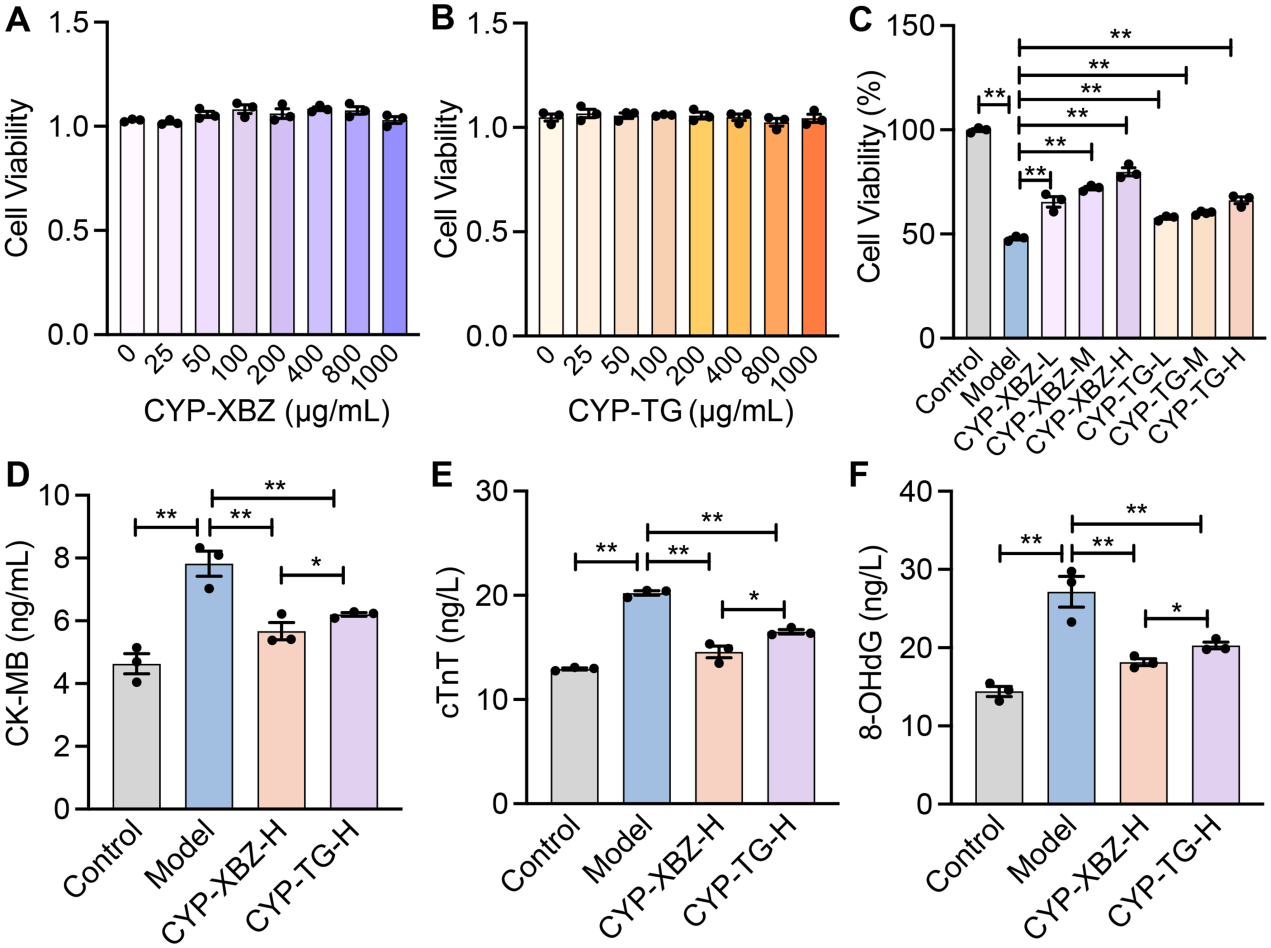
Protective effects of CYP-XBZ and CYP-TG against OGD/R-induced injury in H9c2 cells. **(A,B)** Basal cell viability after 0–1,000 μg/mL treatment. **(C)** Cell viability after OGD/R and polysaccharide treatment. **(D–F)** CK-MB, cTnT, and 8-OHdG levels. Data are mean ± SEM (*n* = 3 independent biological replicates). *p < 0.05, **p < 0.01.

To determine whether CYP-XBZ and CYP-TG exert protective effects under hypoxia–reoxygenation stress, H9c2 cardiomyocytes were subjected to OGD/R treatment with or without polysaccharide intervention. Neither CYP-XBZ nor CYP-TG affected basal cell viability at concentrations up to 1,000 μg/mL (Figures 3A,B), indicating good cytocompatibility within the tested range. OGD/R exposure markedly reduced cell viability compared with the control group (*p* < 0.01), confirming successful establishment of the injury model (Figure 3C). Treatment with CYP-XBZ or CYP-TG significantly improved cell viability in a dose-dependent manner between 25 and 100 μg/mL. At 100 μg/mL, both fractions restored viability to approximately 70%–80% of control levels. Consistent with viability data, OGD/R significantly increased CK-MB and cTnT release (Figures 3D,E), indicating cardiomyocyte membrane damage. In parallel, 8-OHdG levels were markedly elevated (Figure 3F), reflecting oxidative DNA injury. High-dose CYP-XBZ and CYP-TG significantly reduced CK-MB, cTnT, and 8-OHdG levels compared with the model group (*p < 0.05 or **p < 0.01). The magnitude of reduction was similar between the two fractions, with only minor differences observed. These findings demonstrate that both polysaccharides attenuate OGD/R-induced cytotoxicity and biochemical indicators of myocardial injury.

### 3.4 Transcriptomic analysis reveals coordinated regulation of stress-responsive pathways

**Figure 4:**
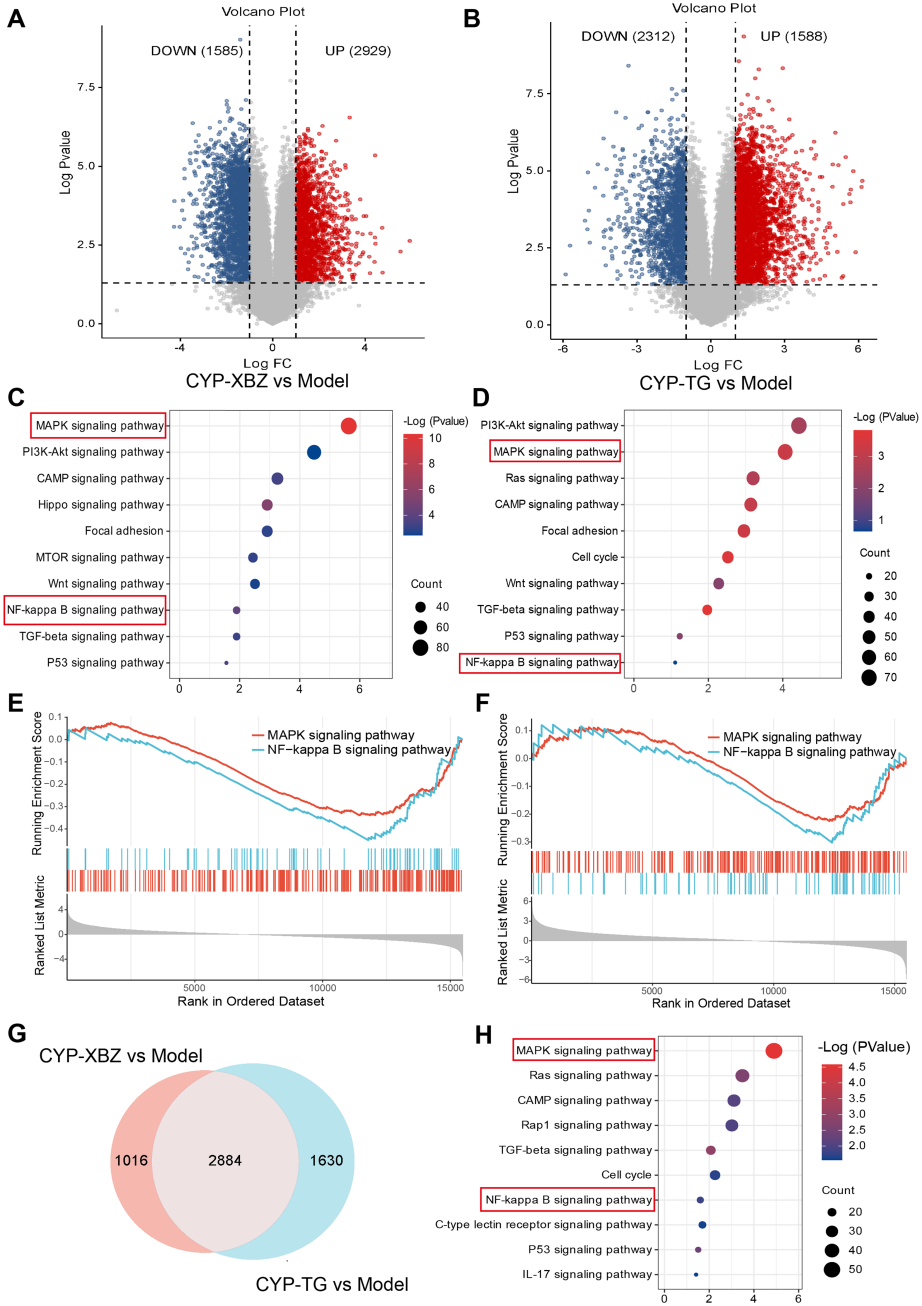
Transcriptomic profiling and pathway enrichment analysis of CYP-treated H9c2 cells under OGD/R conditions. **(A,B)** Volcano plots of DEGs. **(C,D)** KEGG enrichment analyses. **(E,F)** GSEA of MAPK and NF-κB signaling pathways. **(G)** Overlapping DEGs. **(H)** KEGG enrichment analysis of shared DEGs. DEGs were defined as |log₂ fold change| > 1 and adjusted *p* < 0.05.

Transcriptomic analysis was performed to investigate molecular responses associated with cytoprotection. Differential expression analysis identified extensive transcriptional changes in both treatment groups compared with the model group (Figures 4A,B). CYP-XBZ treatment resulted in 2929 up-regulated and 1,585 down-regulated genes. CYP-TG treatment yielded 1,588 up-regulated and 2,312 down-regulated genes. KEGG enrichment analysis showed that DEGs were mainly associated with stress and inflammation-related signaling pathways (Figures 4C,D). The MAPK signaling pathway was significantly enriched in both comparisons. The NF-κB pathway was also identified. Additional enrichment was observed in PI3K-Akt, Ras, and cAMP pathways. GSEA analysis confirmed enrichment of MAPK and NF-κB gene sets in both treatment groups (Figures 4E,F). Intersection analysis revealed 2,884 shared DEGs between CYP-XBZ and CYP-TG treatments (Figure 4G). KEGG analysis of the overlapping genes again highlighted MAPK and NF-κB pathways (Figure 4H). These results indicate highly similar transcriptional responses induced by the two polysaccharide fractions.

### 3.5 Proteomic analysis indicates convergent modulation of MAPK-centered networks

**Figure 5:**
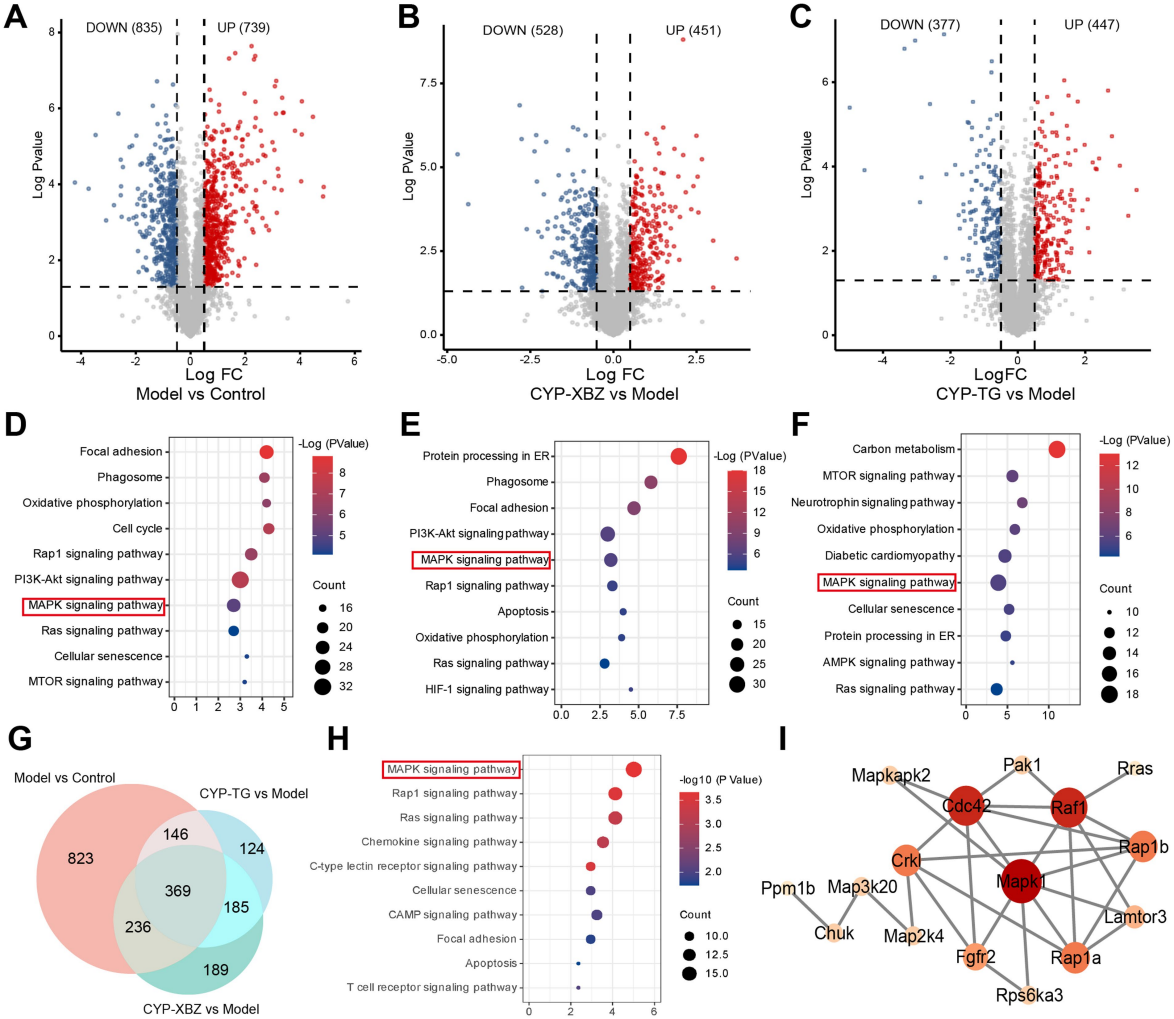
Proteomic profiling and pathway enrichment analysis in OGD/R-treated H9c2 cells. **(A–C)** Volcano plots of DEPs. **(D–F)** KEGG enrichment analyses. **(G)** Overlapping DEPs. **(H)** KEGG enrichment analysis of shared DEPs. **(I)** PPI network of MAPK-related proteins. DEPs were defined as |log₂FC| > 0.26 and adjusted *p* < 0.05.

Quantitative proteomic analysis was conducted to determine whether transcriptional changes were reflected at the protein level. Using |log₂FC| > 0.26 and adjusted *p* < 0.05 as criteria, 451 up-regulated and 528 down-regulated proteins were identified in the CYP-XBZ vs. Model comparison. In the CYP-TG vs. Model comparison, 447 proteins were up-regulated and 377 were down-regulated (Figures 5A–C). KEGG enrichment analysis indicated that DEPs were associated with MAPK signaling, Ras signaling, and oxidative phosphorylation (Figures 5E,F). These pathways are closely related to stress adaptation and energy metabolism under hypoxic conditions. Intersection analysis identified 369 overlapping proteins among comparisons (Figure 5G). Enrichment analysis of this shared subset again highlighted MAPK signaling as a significantly represented pathway (Figure 5H). To further characterize pathway-level interactions, a PPI network was constructed for MAPK-associated proteins derived from overlapping DEPs (Figure 5I). Within this network, MAPK1 exhibited relatively high connectivity, suggesting a central position in the interaction framework. These results indicate that CYP-XBZ and CYP-TG induce convergent protein-level modulation of MAPK-centered signaling networks under OGD/R conditions.

### 3.6 Biochemical validation of MyD88-associated MAPK/NF-κB signaling

**Figure 6:**
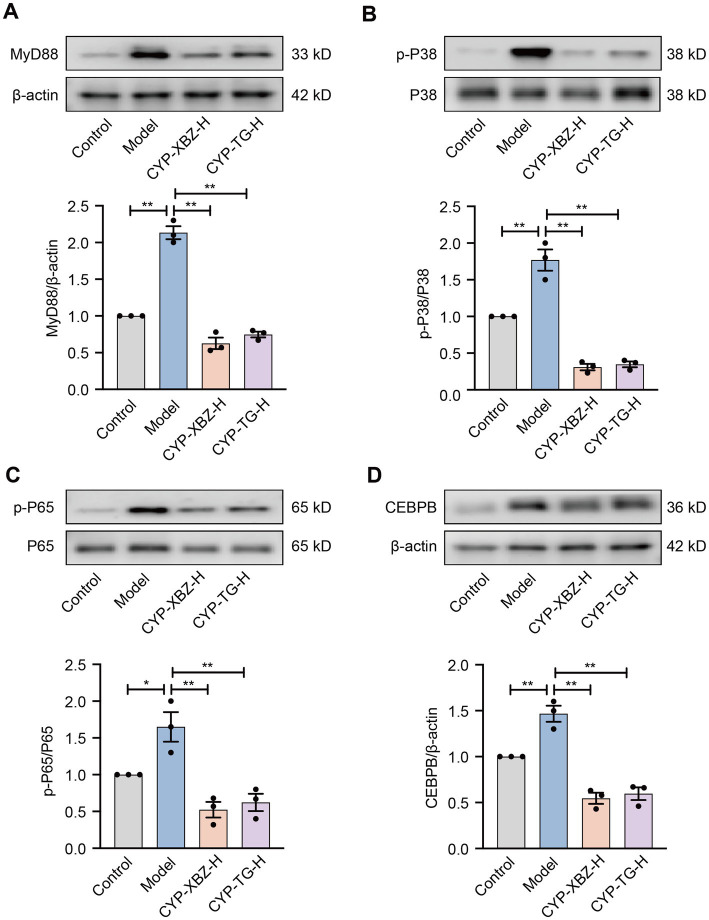
Validation of MAPK/NF-κB signaling modulation by CYP-XBZ and CYP-TG in OGD/R-treated H9c2 cells. Protein bands correspond to MyD88 **(A)**, p-P38/P38 MAPK **(B)**, *p*-P65/P65 **(C)**, CEBPB **(D)**, and *β*-actin. Quantitative analyses were derived from the same biological replicates as the representative blots.

Representative proteins in the MyD88-associated MAPK/NF-κB signaling axis were examined by Western blotting. As shown in Figures 6A–D, OGD/R exposure significantly increased MyD88 and CEBPB protein expression compared with the control group. Phosphorylation levels of P38 MAPK and NF-κB P65 were also elevated, as reflected by increased p-P38/P38 and p-P65/P65 ratios. Treatment with high-dose CYP-XBZ or CYP-TG significantly reduced MyD88 and CEBPB protein expression and decreased phosphorylation ratios of P38 MAPK and NF-κB P65 relative to the model group (*p < 0.05 or **p < 0.01). The overall regulatory patterns were comparable between the two fractions. Under identical experimental conditions, CYP-XBZ showed slightly lower phosphorylation levels of P38 and P65 than CYP-TG, although the differences were modest.

### 3.7 Protective effects of CYP-XBZ and CYP-TG against MI/RI in rats

**Figure 7:**
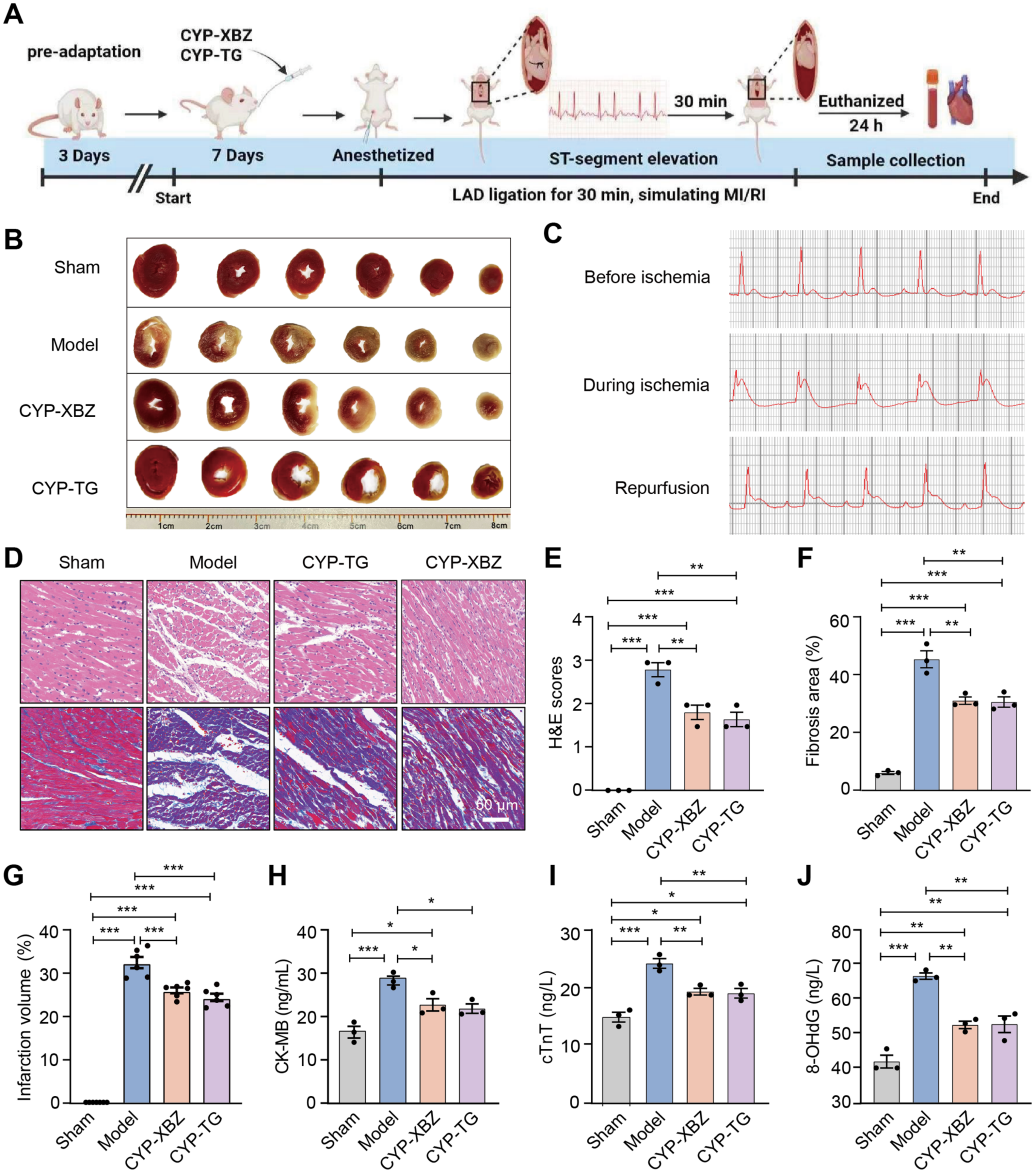
Cardioprotective effects of CYP-XBZ and CYP-TG in a rat model of MI/RI. **(A)** Experimental design. **(B)** TTC-stained heart sections. **(C)** Representative ECG traces. **(D)** H&E and Masson’s trichrome staining. Group order in panel D is Sham, Model, CYP-XBZ, and CYP-TG. **(E–J)** Quantitative analyses of myocardial injury, fibrosis, infarction volume, CK-MB, cTnT, and 8-OHdG. Data are mean ± SEM. *p < 0.05, **p < 0.01, ***p < 0.001. Sample sizes were *n* = 6 for infarction volume and serum biomarkers and *n* = 3 for histology. No animals were excluded from the final analyses.

The effects of CYP-XBZ and CYP-TG were further evaluated in a rat model of MI/RI. The experimental design is illustrated in Figure 7A. TTC staining showed extensive myocardial infarction in the model group and reduced infarction volume in the CYP-XBZ and CYP-TG groups (Figure 7B). Quantitative analysis confirmed a significant decrease in infarction volume in both treatment groups compared with the model group, with no significant difference between CYP-XBZ and CYP-TG (Figure 7G). ECG monitoring showed typical ST-segment elevation during LAD ligation (Figure 7C). H&E staining showed myocardial fiber disorder, inflammatory cell infiltration, and structural disruption in the model group. Masson’s trichrome staining showed collagen deposition and myocardial fibrosis (Figure 7D). Quantitative analysis showed lower myocardial injury scores and fibrosis area in both treatment groups than in the model group (Figures 7E,F). Serum CK-MB, cTnT, and 8-OHdG levels were also reduced by CYP-XBZ or CYP-TG treatment compared with the model group (Figures 7H–J). No significant difference was observed between the two treatment groups for these indicators.

### 3.8 Validation of MyD88-associated MAPK/NF-κB signaling pathway expression *in vivo*

**Figure 8:**
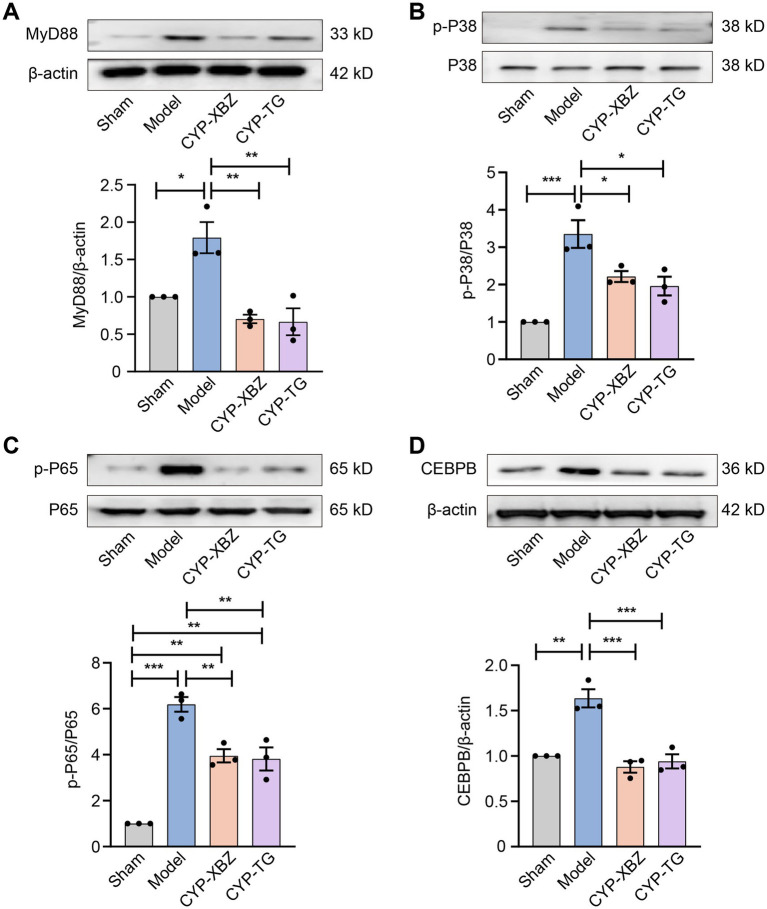
*In vivo* validation of MyD88-associated MAPK/NF-κB signaling modulation by CYP-XBZ and CYP-TG. Protein bands correspond to MyD88 **(A)**, *p*-P38/P38 MAPK **(B)**, p-P65/P65 **(C)**, CEBPB **(D)**, and *β*-actin. Quantitative analyses were derived from the same biological replicates as the representative blots. Data are mean ± SEM (*n* = 3 independent biological replicates). *p < 0.05, **p < 0.01, ***p < 0.001.

To further verify whether the cardioprotective effects observed *in vivo* involve the MAPK/NF-κB signaling pathway, the expression of key proteins was examined in myocardial tissues by Western blotting analysis. Compared with the sham group, MI/RI markedly increased the expression of MyD88 and CEBPB proteins in myocardial tissues (Figures 8A,D), indicating activation of stress and inflammation-related signaling pathways. In parallel, phosphorylation levels of P38 MAPK and NF-κB P65 were significantly elevated in the model group, as reflected by increased p-P38/P38 and p-P65/P65 ratios (Figures 8B,C). Treatment with CYP-XBZ or CYP-TG significantly reduced the expression of MyD88 and CEBPB and suppressed the phosphorylation of P38 MAPK and NF-κB P65 compared with the model group. These findings are consistent with the results obtained in the cellular OGD/R model and indicate that CYP-XBZ and CYP-TG attenuate MI/RI by modulating the MyD88-mediated MAPK/NF-κB signaling pathway *in vivo*.

## 4 Discussion

Plant-derived polysaccharides are receiving increasing attention as natural functional components for nutrition and health-related applications (36–38). Chinese yam polysaccharides are particularly attractive because Chinese yam is both a widely consumed crop and a traditional food–medicine homologous resource (39, 40). In this study, we focused not only on biological activity but also on cultivar comparability, because the stability of functional traits across major edible cultivars is important for the development of reliable food-derived functional ingredients. By combining physicochemical characterization, *in vitro* and *in vivo* functional assessment, and multi-omics analysis, the present work demonstrates that polysaccharides from Xiao-Bai-Zui and Tie-Gun yams share broadly comparable structural features and cardioprotective activities.

The structural characterization results indicate that cultivar variation did not fundamentally alter the core polysaccharide framework. Both CYP-XBZ and CYP-TG exhibited heterogeneous but overlapping molecular weight distributions, similar UV absorption patterns, and closely matched FT–IR spectra, suggesting preservation of the main backbone features and functional groups (41). Monosaccharide analysis further showed that both fractions were dominated by glucose, with only minor differences in galactose and mannose proportions. These findings suggest that the major polysaccharide architecture is largely conserved between the two cultivars, whereas the detected differences are mainly quantitative rather than qualitative. From the perspective of functional ingredient development, this result supports chemical consistency across two major edible Chinese yam cultivars rather than strong cultivar-specific divergence.

Further biological evaluation showed that both polysaccharide fractions effectively alleviated MI/RI both *in vitro* and *in vivo*. In the OGD/R-induced H9c2 cells, CYP-XBZ and CYP-TG improved cell viability and reduced CK-MB, cTnT, and 8-OHdG levels, indicating attenuation of membrane injury and oxidative damage. These effects were accompanied by highly similar transcriptomic and proteomic responses, with both treatment groups showing enrichment of stress and inflammation-related pathways, particularly MAPK and NF-κB signaling pathways. Western blotting validation further demonstrated that both fractions downregulated MyD88 and CEBPB expression and reduced the phosphorylation levels of P38 MAPK and NF-κB P65. Together, these data indicate that the two fractions converge on a common protective mechanism centered on suppression of stress-responsive inflammatory signaling under hypoxia–reoxygenation conditions (42, 43). Importantly, the *in vitro* findings were supported by the rat MI/RI model *in vivo*. Oral administration of either CYP-XBZ or CYP-TG reduced infarct size, improved myocardial histopathology, decreased fibrosis-related changes, and lowered serum CK-MB, cTnT, and 8-OHdG levels. The *in vivo* Western blotting results were consistent with the cellular data and again showed inhibition of MyD88-associated MAPK/NF-κB signaling. The agreement between the two models strengthens the conclusion that the observed effects are not limited to a single experimental system and enhances the translational relevance of the findings in the context of food-derived cardio protective ingredients. Although CYP-XBZ showed a slightly stronger tendency in some readouts, the overall differences between the two fractions were modest. Therefore, the major conclusion is functional comparability rather than superiority of one cultivar over the other.

From the perspective of food-derived functional ingredients, the most important implication of this work is that the two major edible Chinese yam cultivars exhibited substantial consistency at the levels of extractability, structural profile, bioactivity, and signaling response. This consistency is valuable for the development of Chinese yam as a reliable source of bioactive polysaccharides for functional foods and nutraceuticals. It also has practical relevance for nutraceutical standardization, because similar structural and functional properties across cultivars may help reduce raw-material variability and improve reproducibility during product development. In addition, these findings suggest that cultivar selection for Chinese yam polysaccharide-based ingredients may not need to rely solely on one traditional or well-studied cultivar, such as Tie-Gun, but could also include other widely consumed cultivars such as Xiao-Bai-Zui, provided that appropriate quality control standards are applied.

Several limitations should nevertheless be acknowledged. First, the structural characterization in the present study was limited to molecular weight distribution, monosaccharide composition, and spectroscopic features, and did not include detailed linkage analysis, branching patterns, or conformational characterization. Moreover, the polysaccharides used in this study were derived from a single extraction batch per cultivar. Although identical extraction procedures were applied to both cultivars to ensure comparability, future studies involving multiple independent batches are necessary to evaluate batch-to-batch reproducibility. Second, the tested fractions were crude polysaccharides obtained without further fractionation, and minor co-extracted components such as residual proteins may still have contributed to activity. Third, although transcriptomics and proteomics consistently highlighted shared signaling responses, the mechanistic validation focused mainly on the MyD88-associated MAPK/NF-κB axis, and additional pathways identified by omics analysis may also participate in the observed protection. Finally, while the rat MI/RI model strengthened the biological relevance of the findings, further studies on gastrointestinal stability, absorption, metabolism, and long-term efficacy are needed before translating these polysaccharides into food or nutraceutical applications.

Overall, this study demonstrates that polysaccharides from Xiao-Bai-Zui and Tie-Gun Chinese yams exhibit highly comparable structural features, similar protective effects against MI/RI, and convergent regulatory actions on MyD88-mediated MAPK/NF-κB signaling. These findings provide experimental support for the functional consistency of major Chinese yam cultivars and offer a scientific basis for their potential application as food-derived functional ingredients in nutrition and cardiovascular health-related fields.

## 5 Conclusion

This study comparatively evaluated polysaccharides from two major Chinese yam cultivars, Xiao-Bai-Zui and Tie-Gun, using structural characterization, *in vitro* and *in vivo* functional assessment, multi-omics analysis, and pathway validation. Despite minor differences in molecular heterogeneity and monosaccharide composition, the two fractions exhibited highly similar physicochemical features and comparable protective effects against ischemia/reperfusion injury in both cellular and animal models. Their shared bioactivities were associated with suppression of MyD88-mediated MAPK and NF-κB signaling. These findings indicate that cardioprotective function is largely conserved across these two representative cultivars, supporting the functional consistency of important yam germplasm resources. This work provides a scientific basis for developing Chinese yam polysaccharides as consistent sources of food-derived functional ingredients for nutraceutical, cardiovascular, and nutrition-related health applications. Further studies on fine structure, processing suitability, and bioavailability are warranted.

## Data Availability

The original contributions presented in the study are included in the article/Supplementary material, further inquiries can be directed to the author.
